# From Field to Pharmacy: Isolation, Characterization and Tableting Behaviour of Microcrystalline Cellulose from Wheat and Corn Harvest Residues

**DOI:** 10.3390/pharmaceutics16081090

**Published:** 2024-08-20

**Authors:** Djordje Medarević, Maša Čežek, Aleksandar Knežević, Erna Turković, Tanja Barudžija, Stevan Samardžić, Zoran Maksimović

**Affiliations:** 1 Department of Pharmaceutical Technology and Cosmetology, Faculty of Pharmacy, University of Belgrade, Vojvode Stepe 450, 11221 Belgrade, Serbia; 2 Faculty of Biology, University of Belgrade, Studentski Trg 16, 11000 Belgrade, Serbia; knezevica@bio.bg.ac.rs; 3 Vinča Institute of Nuclear Sciences, National Institute of the Republic of Serbia, University of Belgrade, Mike Petrovića Alasa 12–14, 11351 Belgrade, Serbia; 4 Department of Pharmacognosy, Faculty of Pharmacy, University of Belgrade, Vojvode Stepe 450, 11221 Belgrade, Serbia

**Keywords:** microcrystalline cellulose, harvest residues, agricultural waste, tableting properties

## Abstract

A lack of strategies for the utilization of harvest residues (HRs) has led to serious environmental problems due to an accumulation of these residues or their burning in the field. In this study, wheat and corn HRs were used as feedstock for the production of microcrystalline cellulose (MCC) by treatment with 2–8% sodium hydroxide, 10% hydrogen peroxide and further hydrolysis with 1–2 M hydrochloric acid. The changes in the FT-IR spectra and PXRD diffractograms after chemical treatment confirmed the removal of most of the lignin, hemicellulose and amorphous fraction of cellulose. A higher degree of crystallinity was observed for MCC obtained from corn HRs, which was attributed to a more efficient removal of lignin and hemicellulose by a higher sodium hydroxide concentration, which facilitates the dissolution of amorphous cellulose during acid hydrolysis. MCC obtained from HRs exhibited lower bulk density and poorer flow properties but similar or better tableting properties compared to commercial MCC (Ceolus^TM^ PH101). The lower ejection and detachment stress suggests that MCC isolated from HRs requires less lubricant compared to commercial MCC. This study showed that MCC isolated from wheat and corn HRs exhibits comparable tableting behaviour like commercial sample, further supporting this type of agricultural waste utilization.

## 1. Introduction

The rapid growth of the world’s population is accompanied by parallel growth in crop production in order to meet escalating food needs, as well as the demand from industry and livestock farming. Global crop production has increased up to 9.5 billion tonnes, which is a 54% increase in the period 2000–2021 and is predicted to increase further in the following decades [1,2]. Growing crop production has resulted in an enormous amount of agricultural waste, so developing strategies for sustainable management of this waste is of great importance. It is estimated that around 5 billion tonnes of harvest residues (HRs) are generated annually, of which the majority is from corn (1.16 billion tonnes) and wheat (1.14 billion tonnes). Unfortunately, the majority of agricultural waste remains unused, as the costs associated with the collection, transportation and processing of agricultural waste are generally considered to exceed the value of the products obtained [3]. Usually, HRs are left on the field or burnt after harvesting, which leads to air pollution, emission of greenhouse gases and other toxic products and also negatively affects the soil microflora [4,5]. Therefore, isolating the value-added components from agricultural waste is of utmost importance to mitigate the problems caused by the accumulation and improper management of this waste.

Although the composition of HRs varies depending on the species, age of the residues and storage time [6], the main components are cellulose (30–45%), hemicellulose (20–40%) and lignin (10–25%), with smaller amounts of proteins, pectin, sugars, waxes and inorganic minerals [3]. Various value-added compounds have been isolated from wheat and corn HRs, such as cellulose or its derivatives (microcrystalline [7,8,9,10] and nanocrystalline cellulose [11,12,13]), lactic acid [14], glycoside surfactants [15], xylan sulphates [16], carboxymethyl cellulose [17], monosaccharides [18], p-hydroxycinnamic acid esters [19], silicon dioxide [20], etc., while these residues were also used as starting materials for the production of adsorbents [21] and biochar [22].

Since cellulose is the main component of HRs, much attention has been paid in recent years to the development of processes for the isolation of cellulose and products of its further processing, such as microcrystalline (MCC) and nanocrystalline cellulose (NCC), from agricultural waste. Cellulose is the most abundant polymeric compound on Earth and is attractive due to its numerous applications in the pharmaceutical, food, chemical, energy and textile industries or as a feedstock for the production of other valuable compounds [11]. Chemically, cellulose consists of glucopyranose units linked by β-1-4 glycosidic bonds in chains that are stabilized by multiple hydrogen bonds between free OH groups, resulting in a microfibrillar structure. Cellulose microfibrils form a spirally coiled scaffold consisting of crystalline phases spaced out with amorphous segments [11,23]. The main problem in the technological processes for isolating cellulose is the removal of lignin and hemicellulose, which are firmly bound to the cellulose fibrils. This usually requires harsh conditions, such as treatment with strong acids and/or bases, elevated temperatures and/or pressure, with a combination of these treatments being required in most cases [24].

MCC is purified, partially depolymerized cellulose, which is usually obtained by treating cellulose with mineral acids. The acid breaks the β-1-4 glycosidic bonds mostly in the amorphous regions of the cellulose that bind the crystalline segments, resulting in the cleavage of the long cellulose chains. MCC consists mostly of shorter crystalline segments, which remain intact after acid hydrolysis, and a small fraction of amorphous material [25,26]. Since its appearance on the market in 1964, MCC has become an indispensable starting material in the pharmaceutical, cosmetic, food and chemical industries [25,27]. The most common industrial applications of MCC include diluent, binder and adsorbent in pharmaceutical formulations, stabilizer, emulsifier, anti-caking agent and fat substitute in food, gelling agent, stabilizer and suspending agent in the beverage industry and reinforcing agent in various composite materials [28,29]. MCC also has the potential to be used as a dietary fibre in foods, which has a positive effect on the gastrointestinal tract and has potential hypolipidemic and anti-obesity effects [30]. Due to its wide availability, compatibility with most active ingredients, excellent binding properties, self-disintegration ability and low lubricant requirements, MCC is one of the most commonly used excipients in tablet formulations [25].

Wood and cotton are the main feedstocks for the production of cellulose and MCC. However, these raw materials are becoming more expensive and are only available in limited quantities due to high consumption in the furniture, textile and construction industries and the use of wood for heating [26]. This imposes the need to find alternative cheap and widely available sources for production of MCC, such as residues generated after the cultivation and processing of various agricultural crops. The properties of MCC are highly dependent on the properties of the feedstock and the production process. Characterization of obtained MCC usually involves testing of physicochemical properties such as identity, purity, particle size and shape, while material behaviour in the tableting process is rarely evaluated.

Therefore, in this study different procedures were evaluated for preparation of MCC from wheat and corn HRs. Special attention was paid to the functional characterization of the obtained MCC samples by evaluating the tableting behaviour with a dynamic powder compaction analyser.

## 2. Materials and Methods

### 2.1. Materials

Wheat and corn HRs, collected from agricultural land in the vicinity of Belgrade (Resnik, Belgrade, Serbia), were used as starting materials for isolation of MCC. The following chemicals were used for the treatment of HRs: sodium hydroxide (Honeywell, Charlotte, NC, USA), hydrogen peroxide (Pedrogen^TM^ 30%, Honeywell, Charlotte, NC, USA) and hydrochloric acid (Honeywell, Charlotte, NC, USA). All chemicals used for composition analysis of HRs before and after treatment were of analytical or reagent grade.

Commercially available MCC sample (CEOLUS^TM^ PH101, Asahi Kasei, Tokyo, Japan) was used for comparative evaluation of characteristics important in tableting process of MCC isolated from HRs.

### 2.2. Methods

#### 2.2.1. Isolation of MCC from HRs

Wheat and corn HRs, previously subjected to extraction with hexane and ethanol in order to extract less polar secondary plant metabolites with potential application in the pharmaceutical, cosmetic and food industries, were used as feedstock for isolation of MCC. Milled and sieved plant material was first subjected to treatment with different concentrations of sodium hydroxide (2–8% *w*/*w*) in solid/liquid ratio of 1:30 with boiling under reflux for 2 h. In the alternative treatment, HRs were mixed with 1% sodium hydroxide solution in 1:30 solid/liquid ratio and subjected to heating in the autoclave at temperature of 121 °C and pressure of 1 bar for 1 h. After treatment by either of these methods, the remaining solid material was filtered and rinsed with distilled water until neutral reaction. The collected material was then dried overnight at 65 °C and milled after drying. The material obtained after the first phase was then bleached with a 10% (*w*/*w*) hydrogen peroxide solution in a 1:30 solid/liquid ratio at a pH ~ 10 adjusted by sodium hydroxide. The bleaching procedure lasted for 15 min after boiling. The bleached material was separated from the liquid phase by filtration, rinsed several times with distilled water and dried overnight at 65 °C.

The appropriate treatment method for removal of lignin and hemicellulose, which was applied for MCC isolation, was selected based on the results of analysing the composition of HRs before and after treatment. The material treated with the selected method was then subjected to hydrolysis with different concentrations of hydrochloric acid (1 M, 1.5 M or 2 M) at 75 °C for 90 min. After filtration and rinsing with distilled water, the product obtained was dried overnight at 65 °C and ground in a coffee grinder for 2 min.

#### 2.2.2. Compositional Analysis of HRs before and after Treatment

##### Determination of Hemicellulose Content

Hemicellulose content was determined according to modified Van Soest method [31,32]. A mixture of 1.0 g of crushed dry sample, neutral detergent solution (NDS) (containing EDTA—18.6 g/L; SDS—30.0 g/L; 2-ethoxyethanol—10.0 mL; NaH_2_PO_4_ × H_2_O—4.56 g/L; Na_2_B_4_O_7_ × 10H_2_O—6.81 g/L; pH 6.9–7.1), 0.5 g of Na_2_SO_3_ and a few drops of 1-octanol was heated to boiling for one hour to remove soluble sugars, proteins, pectin, lipids and vitamins from the sample. Sample was further filtered, rinsed three times with boiled water and twice with cold acetone and then dried at 105 °C for 8 h. Sample mass after this treatment and drying was denoted as neutral detergent fibre (NDF). In the next step, NDFs were treated with boiled acid detergent solution (ADS) (CTAB—20.0 g dissolved in 1000.0 mL 0.5 M H_2_SO_4_; pH 6.9–7.1) for 1 h under reflux to remove hemicellulose. The sample was further filtered and rinsed several times with boiled water and acetone and dried overnight at 105 °C, giving a final mass denoted as acid detergent fibre (ADF). Hemicellulose content was calculated as NDF—ADF.

##### Determination of Cellulose and Lignin Content

ADFs were used for further determination of cellulose and lignin content. Lignin content was determined according to the Klasson method [33]. Samples were treated with 72% sulphuric acid (1 mL of 72% sulphuric acid was added per 100 mg of the sample) and incubated in water bath at constant temperature (30 ± 0.5 °C) for 1 h with constant stirring and dilution with water. Samples were further subjected to hydrolysis in autoclave at 121 °C for 1 h followed by filtration and rinsing with boiled water. Filtrate was dried at 105 °C to constant mass, which was denoted as lignin content (LC). Cellulose content was calculated as difference between ADF and LC (ADF—LC).

##### Determination of the Ash Content

After drying of HRs at 105 °C to constant mass, samples were annealed in an annealing furnace (550 ± 50 °C) for 12 h. The residue after annealing is expressed as the ash content in percent relative to the mass of the dried sample.

#### 2.2.3. Physicochemical Characterization of MCC

##### Particle Size Analysis

Particle size of obtained MCC samples was estimated by microscopic analysis of MCC dispersion in silicone oil. Prepared samples were analysed using Olympus BX53P polarizing microscope and cellSens Entry software, version 1.15 (Olympus, Tokyo, Japan). At least 100 particles were measured. Due to elongated shape of particles in analysed samples, maximum length was measured for each particle. The results were expressed as D_10_, D_50_, D_90_ and Span.

##### Fourier-Transform Infrared (FT-IR) Spectroscopy

FT-IR spectroscopy was used to evaluate presence of absorption bands characteristic for MCC in the spectra of MCC samples obtained from HRs and to identify potential differences relative to the spectrum of commercial sample. Analyses were performed using a Nicolet iS10 FT-IR spectrometer (Thermo Scientific, Waltham, MA, USA), equipped with an ATR system (Smart iTR, Thermo Scientific, Waltham, MA, USA). The spectra of the tested samples were collected in the interval from 4000 to 650 cm^−1^, with a resolution of 4 cm^−1^, whereby 16 scans were performed for each spectrum.

##### Powder X-ray Diffraction (PXRD) Analysis

PXRD measurements were performed on a SmartLab Rigaku powder diffractometer (Rigaku, Tokyo, Japan) using Bragg–Brentano geometry. The device utilized a copper anticathode X-ray tube, emitting CuKα radiation with a wavelength of λ = 1.54178 Å. The X-ray tube was operated at 40 kV and 30 mA. Measurements were taken over a 2θ diffraction angle range from 5° to 45°, with a step size of 0.02° and a speed of 2°/min. Crystallinity of analysed samples was estimated based on crystallinity index (CrI), calculated according to the method proposed by Segal et al. [34].

##### Differential Scanning Calorimetry (DSC)

DSC analysis was conducted using a DSC1 instrument (Mettler Toledo, Greifensee, Switzerland) within a temperature range of 25–450 °C. Approximately 5–10 mg of the sample was accurately weighed into 40 μL aluminium pans with pierced lids and heated at a rate of 10 °C/min, under a nitrogen gas flow of 50 mL/min. An empty 40 μL aluminium pan served as the reference. The data collected were analysed using STARe Software, version 12.10 (Mettler Toledo, Greifensee, Switzerland).

#### 2.2.4. Functional Characterization of MCC

##### Determination of Bulk and Tapped Density

The bulk density of the material was determined by calculating the ratio of the mass to the volume occupied by the measured mass of the powder in a 25 mL measuring cylinder. The tapped density was determined as the ratio of the sample mass to the volume recorded after 1250 taps using a Stampfvolumeter STAV2003 instrument (J. Engelsmann AG, Ludwigshafen, Germany). Compressibility index (CI) and Hausner ratio were calculated according to Equations (1) and (2), respectively:

CI (%) = 100 × (tapped density − bulk density)/tapped density(1)

Hausner ratio = tapped density/bulk density(2)

Powder flowability was estimated according to descriptive criteria given in the chapter 2.9.36. of the *European Pharmacopoeia* 11.0 [35].

##### Evaluation of Powder Behaviour in Tableting Process

Gamlen D500 dynamic powder compaction analyser (Gamlen Instruments, Biocity Nottingham, UK) was used to analyse tableting behaviour of MCC samples. A powder mass of ~60 mg was manually filled into the die and subjected to compression by flat-faced 6 mm punch at compression speed of 60 mm/min. Compression of the powder samples was performed in the range of 100–500 kg compression loads, corresponding to compression pressures 34.7 to 173.5 MPa. After compression, the die was rotated, and the tablet was detached from the die plate. In the last ejection phase, the tablet was pushed through the bottom of the die in the tablet holder and subsequently removed from the instrument.

The instrument was operated by the software, and in each phase of powder compaction load vs. punch position curves were generated. The following parameters were used for characterization of powder behaviour during compaction: net work of the compression, in-die elastic recovery, ejection stress, detachment stress and tensile strength. Total work of compression was calculated as an area under the force vs. displacement curve in the compression phase. The net work of compression (NWC) was calculated by subtracting work of elastic recovery from the total work of compression. In-die elastic recovery (IER) as an indicator of powder elastic properties was calculated according to the following equation:

IER (%) = 100 × (t_max_ − t_min_)/t_min_ (3)

where *t_max_* is the tablet thickness at the end of compression phase and is calculated as the difference between base punch position and punch displacement at the end of the compression, which corresponds to zero compression load; *t_min_* is the tablet thickness which corresponds to maximum punch displacement in the compression phase, calculated as the difference between base punch position and maximum punch displacement during compression.

Powder adhesion to die base and walls, which indicates material lubricant properties, was assessed by parameters detachment stress (DS) and ejection stress (ES), calculated according to the following equations:

DS = F_d_/(D/2)^2^ × π (4)

where F_d_ is maximum force recorded during tablet detachment, and D is tablet diameter.

ES = F_e_/π × D × t (5)

Here, F_e_ is maximum force recorded during tablet ejection, D is tablet diameter and t is tablet thickness.

Tablet diameter (D), thickness (t) and breaking force (F) were determined immediately after powder compaction. Tablet breaking force (F) and diameter (D) were determined using Erweka TBH 125D tablet hardness tester (Erweka, Heusenstamm, Germany), while the thickness (t) of the tablets was determined by the digital calliper. The determined parameters were used for the calculation of tablet tensile strength (TS), according to the following equation [36]:

TS = 2F/π × D × t(6)

The calculated parameters used for evaluation of powder tableting behaviour were expressed as mean ± standard deviation of four replicates.

## 3. Results and Discussion

### 3.1. Compositional Analysis of HRs before and after Treatment

The results of analysing the composition of HRs before and after treatment with sodium hydroxide and hydrogen peroxide are shown in Table 1. The analysis of wheat HRs revealed a higher cellulose and lower hemicellulose content compared to the results of previous studies which demonstrate the following composition: 40–43% cellulose, 32–34% hemicellulose and 14–22% lignin [37,38]. On the other hand, the cellulose content in corn HRs was slightly lower compared to the literature data for corn stover, which reported 50–55% cellulose, 39.39% hemicellulose and 7.5% lignin [39]. Based on the results obtained, wheat HRs appears to be the more suitable feedstock for the production of MCC, but the cellulose content in corn HRs is also sufficiently high to justify its commercial use as a cellulose source. It is also important to keep in mind that the composition of cereal HRs is significantly influenced by climatic conditions, species variety, harvest time and soil properties [40]. Chemical treatments with different concentrations of sodium hydroxide and subsequent bleaching with hydrogen peroxide led to a significant reduction in lignin and hemicellulose content. Treatment with 1% sodium hydroxide solution at elevated temperature and pressure in an autoclave did not show higher efficiency in the removal of lignin and hemicellulose. However, the application of such more extreme conditions may be useful in reducing the consumption of chemicals in the treatment process and amount of generated chemical waste. Based on the compositional analysis, the treatment methods that yielded the highest cellulose content were selected for the isolation of MCC. Therefore, 4% and 8% sodium hydroxide solutions were used for the treatment of wheat and corn HRs, respectively, followed by bleaching with a 10% hydrogen peroxide solution. After this treatment, samples were subjected to acid hydrolysis with 1, 1.5 and 2 M hydrochloric acid (Table 2).

### 3.2. Physicochemical Characterization of MCC

#### 3.2.1. Microscopic Analysis

**Figure 1:**
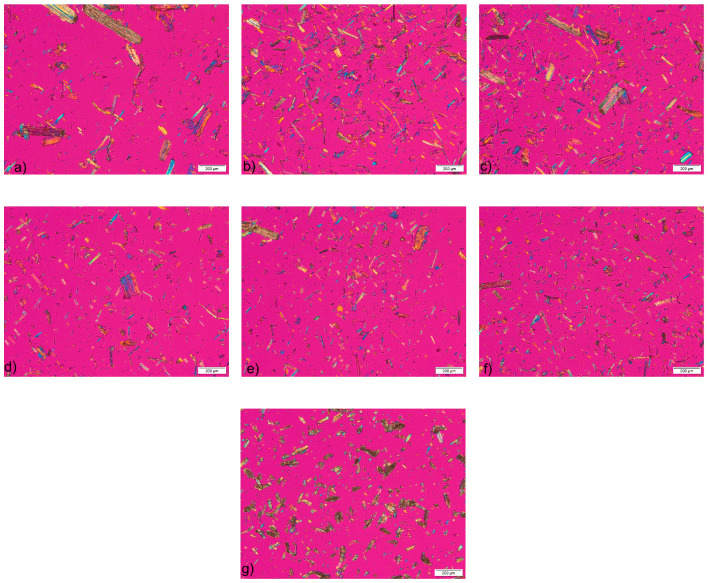
Micrographs of samples (**a**) Wa, (**b**) Wb, (**c**) Wc, (**d**) Ca, (**e**) Cb, (**f**) Cc and (**g**) Ceolus^TM^ PH101 observed under a polarizing microscope.

The photomicrographs of the samples, obtained by polarized light microscopy, show the fibrillar morphology of the MCC particles (Figure 1). The presence of birefringence confirmed the highly crystalline nature of the MCC samples obtained from HRs, which can be attributed to the successful removal of the largest part of the amorphous fraction by acid hydrolysis. The slightly lower birefringence observed on the micrographs of the commercial MCC sample indicates a lower degree of crystallinity.

The microscopic analysis showed broad particle size distribution for both MCC samples obtained from HRs and commercial MCC. This was confirmed by the results of particle size measurement on the photomicrographs (Table 3). The mean particle size of the Ceolus^TM^ PH101 sample is very close to 50 μm, which is stated by the manufacturer in the product information [41]. The particle sizes for all MCC samples obtained by chemical treatment of the HRs are within the range calculated for commercial sample. There is a tendency for the particle size to decrease with increasing acid concentration, which is due to more efficient hydrolysis of the glycosidic bonds in the cellulose chains. From the calculated span values, it can be concluded that all samples exhibit a heterogeneous particle size distribution, which is particularly pronounced in the samples obtained from wheat HRs. A heterogeneous particle size distribution is a typical characteristic of cellulosic materials [28]. From the results of the microscopic analysis, it can be summarized that the MCC samples obtained from HRs, although obtained by a simple laboratory isolation procedure, have similar morphological characteristics to commercial MCC.

#### 3.2.2. FT-IR Spectroscopy

**Figure 2:**
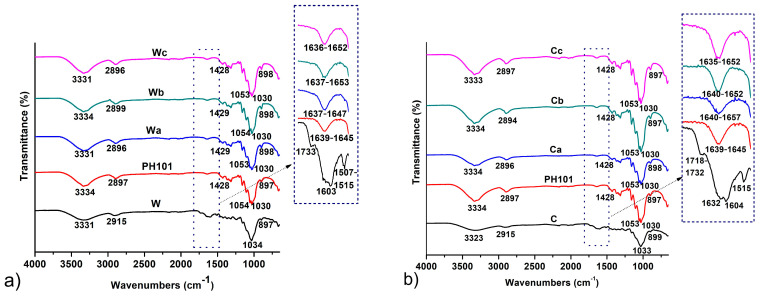
FT-IR spectra of MCC samples obtained from (**a**) wheat (Wa-Wb) and (**b**) corn (Ca-Cb) harvest residues in comparison with initial wheat (W) and corn (C) harvest residues and a commercial Ceolus^TM^ PH101 (PH101) sample.

Very similar FT-IR spectra were obtained for both MCC samples prepared from wheat and corn HRs, which closely matched the spectrum of the Ceolus^TM^ PH101 sample (Figure 2). The characteristic absorption bands at 3331–3334 cm^−1^ and 2894–2899 cm^−1^ correspond to the stretching vibrations of hydrogen-bonded primary and secondary OH groups and aliphatic C-H groups, respectively [11,24]. The broad peaks observed at ~1640 cm^−1^ result from the bending vibrations of the OH groups of water molecules absorbed on the cellulose surface [29,42]. The peak at 1428 cm^−1^ is attributed to the bending vibrations of CH_2_ groups and is commonly considered an indicator of the crystallinity of cellulose products [28,29]. Absorption bands at 1030 cm^−1^ and 1053–1054 cm^−1^ correspond to the stretching vibrations of C-O-C ether bonds [43]. Peaks at 897–898 cm^−1^ are associated with stretching C-O-C vibrations in β-1-4 glycosidic bonds in cellulose chains [44]. To evaluate the changes that occur after chemical treatment, the spectra of HRs as starting materials were compared with the spectra of the obtained MCC samples. The peaks at 3331 cm^−1^ and 3323 cm^−1^ characteristic of the stretching vibrations of OH groups for wheat and corn HRs became sharper and decreased in intensity after chemical treatment, which is due to the disruption of hydrogen bonds between cellulose and non-cellulosic compounds as well as between cellulose chains, resulting in an increase in the proportion of free OH groups [8,24]. The successful removal of hemicellulose and lignin is also confirmed by the complete disappearance after chemical treatment of the weak, broad absorption bands between 1718 and 1733 cm^−1^, reflecting the C=O stretching vibration of the carbonyl and acetyl groups in the xylan component of hemicellulose and the carbonyl ester of the monomeric unit of p-coumaric acid in lignin [44,45]. Moreover, peaks positioned at 1507–1515 cm^−1^ and 1603–1604 cm^−1^ in the spectra of HRs, which are assigned to the symmetric in-plane stretching vibrations of the C=C groups of the aromatic ring in lignin [44,46], disappeared after chemical treatment. Absorption bands at 1242 cm^−1^ assigned to the C-O-C vibration of the aryl-alkyl ether in lignin molecules [45] are also absent in the spectra of the MCC molecules, confirming the successful removal of lignin. The observed changes in the FT-IR spectra after the chemical treatment of HRs in the MCC production process confirmed that the applied treatment method successfully removed most of the lignin and hemicellulose from the starting material.

#### 3.2.3. PXRD Analysis

**Figure 3:**
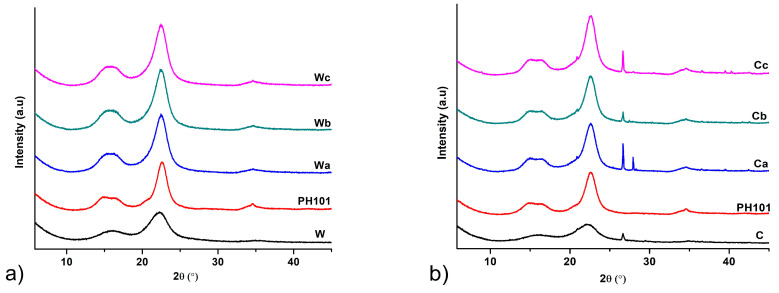
PXRD diffractograms of MCC samples obtained from (**a**) wheat (Wa-Wb) and (**b**) corn (Ca-Cb) harvest residues in comparison with starting wheat (W) and corn (C) harvest residues and commercial Ceolus^TM^ PH101 (PH101) sample.

All MCC samples analysed exhibited a PXRD pattern characteristic of native cellulose I. The diffractograms obtained by PXRD analysis with peaks at approximately 15, 16.5, 22.5 and 34.5° *2*θ which correspond to crystal planes 101, 10$\bar{1},$ 002 and 040 (Figure 3) are consistent with those previously reported in the literature for cellulose I [47]. An additional peak at 26.7° *2*θ can be observed in the diffractograms of the samples obtained by treating corn HRs. A similar peak was also reported in the literature for MCC obtained from *Posidonia oceanica* brown algae as feedstock material [47]. This peak is also seen in the PXRD pattern of corn HRs and probably originates from the mineral impurities present. The slightly higher ash content found in the analysis of these samples (Table 1) indicates a higher proportion of mineral impurities in the corn HRs and the resulting MCC samples. PXRD analysis showed that the method used for isolation of MCC in this study did not induce transformation from cellulose I to cellulose II. This polymorphic transition has been previously reported in the literature for MCC obtained from corn cobs, as the treatment of the plant material was performed with a high sodium hydroxide concentration (17.5%). Treatment processes with sodium hydroxide concentrations below 10% generally do not lead to polymorphic transitions of the cellulose [10].

It is clearly evident that the diffraction peaks observed in the PXRD patterns of HRs become sharper after chemical treatment in the MCC isolation procedure due to the removal of lignin, hemicellulose and amorphous cellulose fraction. The glycosidic bonds in the amorphous cellulose fraction are more susceptible to cleavage upon acid treatment, leading to the dissolution of this fraction and an increase in the crystallinity of the product [24]. This is confirmed by the significant increase in CrI, an indicator of crystallinity, after chemical treatment. All MCC samples obtained from HRs showed a CrI value above 75%, which is slightly higher compared to the commercial Ceolus^TM^ PH101 sample. This confirms that the chemical treatment applied in this study successfully removed the non-cellulosic components and part of the amorphous cellulose fraction. Although several studies have found that the degree of crystallinity of MCC depends more on the starting material than on the processing conditions [48,49], our results did not confirm this hypothesis. The calculated CrI values for MCC isolated from corn HRs were higher than those of MCC isolated from wheat HRs, although the CrI value of wheat HRs was 10% higher than that of corn HRs. From the results of compositional analysis, it appears that the treatment with 8% sodium hydroxide removes a greater amount of lignin from corn HRs than the treatment with 4% sodium hydroxide that was used for wheat HRs, which facilitates the dissolution of the amorphous cellulose fraction in the acid treatment step. An increase in the CrI value is usually associated with an improvement in the mechanical properties and thermal stability of cellulose derivatives [50]. Alemdar and Sain reported a similar CrI value (77.8%) for cellulose nanofibers isolated from wheat straw [38]. The CrI values for MCC from corn HRs were similar to those reported by Azubuike and Okhamafe for MCC from corn cobs [10] and higher than those reported by Jantip and Suwanruji for MCC from corn husks and corncobs [51], although these studies used more complex treatment procedures involving higher sodium hydroxide concentrations and/or bleaching with less environmentally friendly sodium hypochlorite. Singh et al. obtained MCC with higher CrI from corn stover but using a treatment procedure involving prolonged exposure to sodium hydroxide, further treatment with sodium chlorite and hydrolysis with 10% sulphuric acid [8].

#### 3.2.4. DSC Analysis

**Figure 4:**
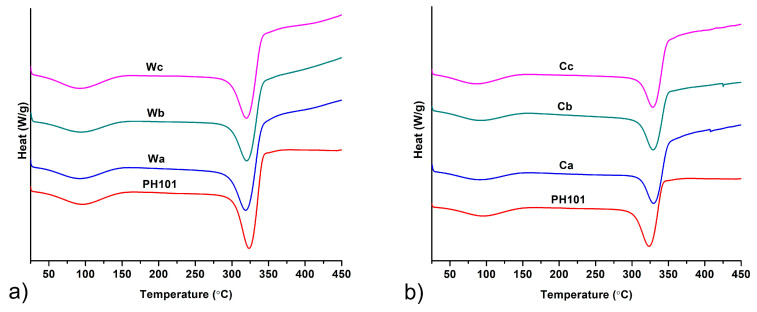
DSC thermograms of MCC samples obtained from (**a**) wheat (Wa-Wb) and (**b**) corn (Ca-Cb) harvest residues in comparison with commercial Ceolus^TM^ PH101 (PH101) sample.

DSC thermograms of MCC obtained from wheat and corn HRs in comparison to a commercial Ceolus^TM^ PH101 sample are shown on Figure 4. Very similar thermograms were obtained by heating of all samples, which comply with MCC thermograms reported in the literature [10,28,52]. A very broad endotherm positioned between ~60 °C and ~140 °C corresponds to evaporation of absorbed water molecules [28]. The second thermal event observed on the thermograms was a peak at 323.5 °C for Ceolus^TM^ PH101, 318.4–320.2 °C and 328.5–329.7 °C for MCC obtained from wheat and corn HRs, respectively. These peaks occurred due to thermal decomposition of the cellulose backbone [53]. The slightly higher thermal stability of MCC obtained from corn HRs is in accordance with its higher degree of crystallinity, determined by PXRD, as the breaking of chemical bonds in the cellulose backbones is much easier in the amorphous fraction [54].

### 3.3. Functional Characterization of MCC

#### 3.3.1. Powder Flow Properties

MCC samples obtained from HRs exhibited lower bulk and tapped density compared to commercial MCC samples (Table 4). It can be observed that samples Ca-Cc exhibited higher bulk and tapped densities compared to those isolated from wheat HRs. This correlates with the higher degree of crystallinity of the samples obtained from corn HRs and results from the higher extent of removal of the voluminous amorphous fraction. The bulk and tapped densities determined correspond to the literature data for MCC isolated from corn husks [52] and are significantly higher than the values previously reported for MCC isolated from wheat straw [7]. Descriptive classification of flowability based on the CI and Hausner ratio confirmed the poor flow properties of MCC samples obtained from both wheat and corn HRs (Table 4). Commercial MCC exhibited better flow properties, as expected due to the highly controlled industrial production process, which usually involves spray drying. Low bulk density and poor flow properties are well-known characteristics of MCC caused by a wide particle size distribution and an irregular and elongated particle shape. This often imposes the need for particle engineering of MCC or combination with free-flowing excipients in order to achieve the characteristics of the powder mixture required for the production of tablets by direct compression. However, the low bulk density and broad particle size distribution allow for favourable powder compression behaviour and a very high dilution potential of MCC.

#### 3.3.2. Evaluation of Powder Behaviour in Tableting Process

**Figure 5:**
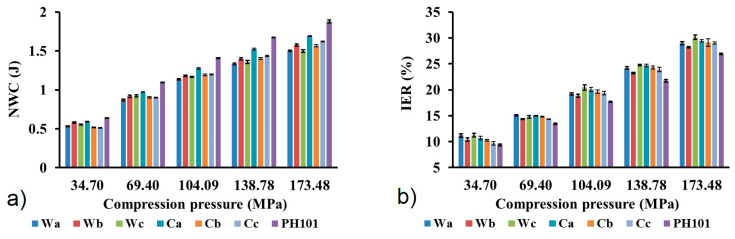
(**a**) Net work of compression (NWC) and (**b**) in-die elastic recovery (IER) of MCC samples obtained from wheat (Wa-Wb) and corn (Ca-Cb) harvest residues and commercial Ceolus^TM^ PH101 (PH101) sample as a function of compression pressure.

Similar NWC values were calculated for MCC samples obtained from wheat and corn HRs, while slightly higher values were observed for the commercial sample (Figure 5a). The NWC values increase with increasing compression pressure due to the higher energy input, which results in a higher extent of particle deformation [55]. Since MCC deforms predominantly by energy demanding plastic deformation, high NWC values are to be expected. High NWC values are also a consequence of the strong intermolecular hydrogen bonds between the hydroxyl groups in the cellulose structure. Materials that deform plastically release a considerable amount of energy after the compressive force has ceased, which leads to an expansion of the compact, known as elastic relaxation. Elastic relaxation was estimated using the IER values, which represent the extent of the change in compact dimensions after the cessation of compression pressure. There is a clear tendency for the IER to increase with increasing compression pressure (Figure 5b), as the material stores more elastic energy at higher pressure, which is released in the decompression phase. Commercial MCC samples showed only slightly lower IER values at each compression pressure. Although the calculated IER values are in the range of 10–30%, they are significantly lower compared to previously reported values for MCC obtained for wheat and corn HRs [7,56]. Elastic relaxation is one of the main causes of tablet capping (the detachment of an upper or lower end piece from the tablet) and lamination (splitting of tablets into multiple layers), which are among the most common tablet defects [57]. However, no capping, lamination or other visible defects were observed in the tablets prepared from each MCC sample. Since plastic deformation of MCC is a time-dependent process, the elastic component tends to become more pronounced at higher tableting speeds when there is insufficient time for plastic deformation to occur. This should be taken into account when scaling up the tableting process, where the elastic component can be controlled by choosing the appropriate tableting speed and dwell time [25].

**Figure 6:**
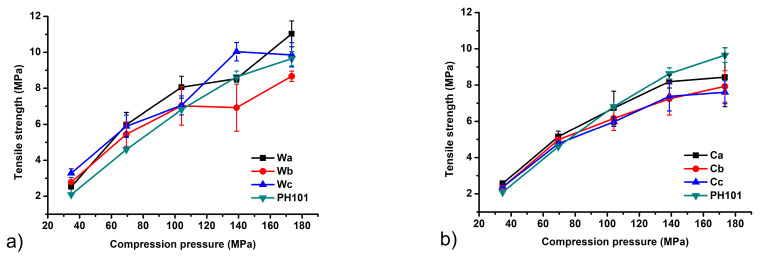
Tablet tensile strength as a function of compression pressure of MCC samples obtained from (**a**) wheat (Wa-Wb) and (**b**) corn (Ca-Cb) harvest residues in comparison with commercial Ceolus^TM^ PH101 (PH101) sample.

The tensile strength (TS) was used as an indicator of the mechanical properties of the tablets prepared from different MCC samples. Tablets with a TS above 2 MPa are considered to have sufficient resistance to withstand stress during the production process, transportation and patient handling [58]. The results obtained show that TS values meeting this criterion are achieved within the entire range of compression pressures used in this study (Figure 6). In general, the TS of all samples increases with increasing compression pressure, as higher compression pressures allow closer packing of the particles and the formation of more interparticle bonds. The only exceptions to this rule were a slight decrease in TS with an increase in compression pressure from 104.1 to 138.8 MPa for sample Wa and 138.8 to 173.5 MPa for sample Wb. A slightly lower TS value was observed for tablets produced with MCC isolated from corn HRs at some compression pressures. The observed differences probably occur due to the different particle size and morphology. MCC is commonly used as a tablet diluent for direct compression due to a higher extent of plastic deformation which results in tablets with very high TS. A larger contact area between the particles due to their elongated shape and the presence of many chemical groups capable of forming intermolecular hydrogen bonds are the underlying mechanisms responsible for the high TS of MCC compacts [25].

**Figure 7:**
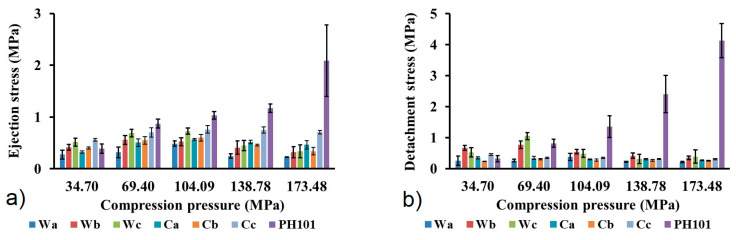
(**a**) Ejection stress and (**b**) detachment stress of MCC samples obtained from wheat (Wa-Wb) and corn (Ca-Cb) harvest residues and commercial Ceolus^TM^ PH101 (PH101) sample as a function of compression pressure.

Ejection stress (ES) and detachment stress (DS) were calculated in order to evaluate the tendency of powder towards sticking for die walls and base. These parameters are useful indicators of material lubricating properties and necessity for addition of lubricant to avoid potential defects in prepared tablets. According to the literature, DS and ES values below 5 MPa are considered acceptable for the production of tablets without defects, such as capping and lamination [59,60]. Due to its extremely low coefficient of friction and very low residual die wall pressure, MCC is characterized with lower lubricant requirements compared to most of tableting excipients, but the addition of lubricant is unavoidable in most cases [25]. Adsorption of hydrophobic lubricants onto surface of powder particles may result in lowering of tablets mechanical resistance and negatively affects powder wetting by the dissolution medium, leading to a lower drug dissolution rate. Such problems more commonly occur for excipients that undergo plastic deformation, such as MCC, while creation of new surfaces during particle fractures reduces this problem for brittle excipients [25]. Very low ES and DS values were calculated for all MCC samples obtained from wheat and corn HRs (Figure 7), indicating good self-lubricating characteristics of the material. The highest value (1.05 MPa) was calculated for DS of the Wc sample at a compression pressure of 69.4 MPa, which is far below previously mentioned literature criteria. Calculated values within the whole range of compression pressures are low, so it is not possible to observe any dependence from the feedstock material, isolation procedure or compression pressure. The commercial MCC sample exhibits higher ES and DS, which is particularly pronounced at the two highest compression pressures used in this study. Although calculated values of ES and DS for the commercial MCC sample are also within the common literature criteria, significantly higher values indicate higher lubricant requirements and the risk of potential problems caused by the addition of lubricants.

## 4. Conclusions

A simple method based on chemical treatment with sodium hydroxide, hydrogen peroxide and hydrochloric acid was efficient in removing hemicellulose and lignin from wheat and corn HRs and resulted in the isolation of MCC. Physicochemical characterization showed very similar properties of the isolated MCC and the commercially available sample. Evaluation of tableting behaviour confirmed that the MCC isolated from wheat and corn HRs had comparable or better properties important for tableting compared to the commercial sample. Significantly lower ejection stress and detachment stress indicates a lower lubricant requirement of isolated MCC. The results of our study show that wheat and corn HRs are very attractive feedstock materials for MCC. The use of HRs for the production of MCC adds value to this waste material and may contribute to the development of strategies for the sustainable management of agricultural waste.

## Figures and Tables

**Table 1:** The results of compositional analysis (CEL—cellulose, HEM—hemicellulose, LIG—lignin) of starting harvest residues and samples subjected to treatment with 1–8% sodium hydroxide and 10% hydrogen peroxide (the samples were labelled with starting material (W—wheat, C—corn) and concentration of sodium hydroxide (1–8%)).

Sample	CEL (%)	HEM (%)	LIG (%)	ASH (%)
W	54.09	23.26	14.11	6.51
C	41.01	37.43	13.17	7.87
W 1%	78.52	10.21	4.62	2.34
W 2%	79.07	11.27	3.14	2.16
W 4%	81.14	8.11	4.2	2.62
W 8%	77.21	8.24	5.58	3.62
C 1%	75.67	13.60	4.12	5.29
C 2%	75.81	12.41	3.71	8.06
C 4%	73.26	15.14	2.62	5.90
C 8%	78.55	12.92	2.56	5.87

**Table 2:** MCC samples obtained from wheat (W) and corn (C) harvest residues (HRs) by hydrolysis with different concentrations (1–2 M) of hydrochloric acid (HCl).

Sample	Source of HRs	HCl Concentration Used for Hydrolysis
Wa	Wheat	1 M
Wb	Wheat	1.5 M
Wc	Wheat	2 M
Ca	Corn	1 M
Cb	Corn	1.5 M
Cc	Corn	2 M

**Table 3:** The results of particle size analysis of MCC samples obtained from wheat (Wa-Wc) and corn (Ca-Cc) harvest residues and commercial Ceolus^TM^ PH101 sample (PH101).

	Wa	Wb	Wc	Ca	Cb	Cc	PH101
Mean particle size (μm)	58.47	49.48	42.97	60.95	45.06	39.05	54.44
D_10_ (μm)	14.92	13.80	9.89	20.47	14.81	14.42	16.07
D_50_ (μm)	35.62	34.89	26.55	50.69	35.59	29.52	43.94
D_90_ (μm)	140.17	99.33	86.13	116.67	81.78	73.75	106.43
Span	3.52	2.45	2.87	1.90	1.88	2.01	2.06

**Table 4:** Bulk and tapped density and flowability classification based on compressibility index (CI) and Hausner ratio for MCC samples obtained from wheat (Wa-Wb) and corn (Ca-Cb) harvest residues in comparison with commercial Ceolus^TM^ PH101 (PH101) sample.

MCC Sample	Bulk Density (g/cm^3^)	Tapped Density (g/cm^3^)	CI (%)	Hausner Ratio	Flowability (Ph. Eur 11.0 (2.9.36))
Wa	0.230 ± 0.009	0.307 ± 0.006	25.01 ± 2.10	1.33 ± 0.04	Poor
Wb	0.209 ± 0.010	0.289 ± 0.017	27.62 ± 0.98	1.38 ± 0.02	Poor
Wc	0.230 ± 0.003	0.361 ± 0.009	36.33 ± 1.56	1.57 ± 0.04	Very poor
Ca	0.236 ± 0.009	0.358 ± 0.015	33.91 ± 0.50	1.51 ± 0.01	Very poor
Cb	0.289 ± 0.001	0.407 ± 0.001	29.13 ± 0.25	1.41 ± 0.01	Poor
Cc	0.324 ± 0.012	0.485 ± 0.030	32.60 ± 1.57	1.48 ± 0.03	Very poor
PH101	0.352 ± 0.016	0.456 ± 0.029	22.67 ± 2.00	1.29 ± 0.03	Passable

## Data Availability

The data presented in this study are available upon request from the corresponding author.
